# Relationships among smoking abstinence self-efficacy, trait coping style and nicotine dependence of smokers in Beijing

**DOI:** 10.18332/tid/125401

**Published:** 2020-09-01

**Authors:** Hanqiao Ma, Xingming Li, Manhua Zhang, Han Liu, Qianying Jin, Kun Qiao, Ali Akbar

**Affiliations:** 1 School of Humanities, Capital Medical University, Beijing, China; 2 School of Public Health, Capital Medical University, Beijing, China

**Keywords:** coping style, nicotine dependence, community, smoking abstinence self-efficacy

## Abstract

**INTRODUCTION:**

Psychological, physiological and social factors play an important role in the initiation, persistence, dependence and relapse of smoking behaviors, and coping style and smoking abstinence self-efficacy can all affect nicotine dependence.

**METHODS:**

A cross-sectional sample of 568 quitters from 19 communities in Beijing in 2019 was surveyed. Demographic information and psychological characteristics of smokers were collected by an interview questionnaire, and psychological traits scales including the Smoking Abstinence Self-Efficacy (SASE) and the Trait Coping Style Questionnaire (TCSQ). We compared differences in psychological traits across demographic information and explored the relationship between nicotine dependence and coping styles and self-efficacy in refusing to smoke.

**RESULTS:**

Significant differences were identified in self-efficacy in refusing to smoke and across dimensions among quitters by gender, job type, education level, and monthly income level (all p<0.05). Males had lower self-efficacy in the habitual/addictive context than females; retirees had better overall self-efficacy and self-efficacy in the negative/emotional context than business service workers and professionals; and high-educated, high-income quitters had lower self-efficacy in the negative/emotional context. There are significant differences in positive coping styles among quitters of different ages, levels of education, and types of work (all p<0.05). The results further showed that the underage population, highly educated population, and practitioners other than those in retirement, are less likely to use positive coping styles. Interventional effects analysis showed that a higher sense of self-efficacy in addictive contexts can counteract some of the negative coping styles that induce smoking.

**CONCLUSIONS:**

Self-efficacy played an indirect mediating role between negative coping style and nicotine dependence; individuals who used more negative coping styles were more likely to engage in smoking and therefore were more nicotine dependent. Hence, it is necessary to reduce the use of negative coping strategies and improve the self-efficacy of smoking abstinence in the face of addiction.

## INTRODUCTION

Smoking is a preventable risk factor for many diseases. It is estimated that, by 2030, eight million people worldwide will die each year from various diseases related to smoking^1^. Although China’s tobacco control has made significant progress, the quitting rate is still low^2^. The 2015 China Adult Tobacco Survey Report showed that 14.4% of all daily smokers had quit smoking, and only 17.6% planned to quit smoking in the next year^3^. Although smokers are aware of the dangers of smoking, they still find it difficult to quit. Smoking and quitting behaviors are complex processes that cannot be explained by a single model, and social, psychological and physiological factors are intertwined and play an important role in the initiation, sustained use, dependence formation and relapse of addictive substances^4^.

Studies have shown that smoking is associated with a number of psychological traits. Smoking abstinence self-efficacy can effectively improve the success of smoking cessation^5,6^. Self-efficacy is smokers’ belief that they can exert control over their smoking activity^7^; so self-efficacy can be considered as an important factor in healthy behavior change^8^. Some studies have shown that there is a significant relationship between smoking abstinence self-efficacy and nicotine dependence^9,10^. Meanwhile, studies have shown that self-efficacy can also affect the use of coping styles^11,12^. In addition, coping style and self-efficacy affect success in quitting smoking^13^, therefore, as an important mediator in the process of psychological stress, coping style is the response of smokers to deal with changes in their internal and external environment that are beyond their capacities. Their positive coping style is characterized by seeking support and changing the value system. In contrast, negative coping is more about avoidance and venting, while the use of negative coping styles will produce more adverse effects on physical and mental health^14-16^ such as the generation of smoking behavior^17^ and failure to quit smoking due to relapse. However, the psychological traits of smokers in Beijing and the relationships among smoking abstinence self-efficacy, trait coping style and nicotine dependence have seldom been studied.

**Figure 1:**
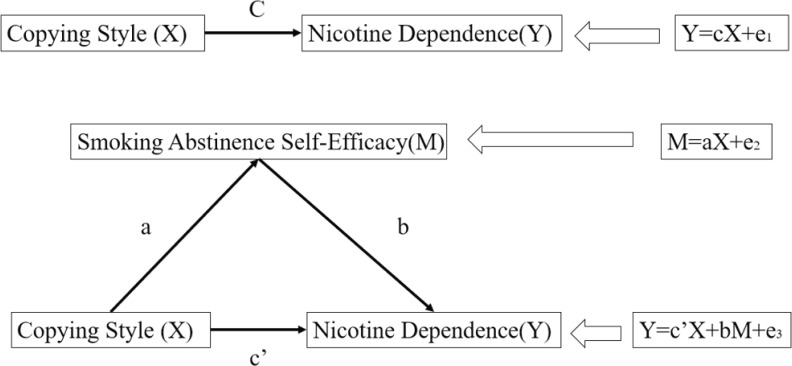
Mediational analysis effect between coping style, nicotine dependence and smoking abstinence self-efficacy

This study proposes the following hypothesis. The use of negative coping styles can lead to increased nicotine dependence on the one hand, while on the other, negative coping styles can lead to a decrease in the smoking abstinence self-efficacy and thus an increase in nicotine dependence. The pathway diagram is shown in Figure 1. In recent years, most research on the relationship between psychological traits and nicotine dependence have focused on the relationship between a single trait and nicotine dependence, therefore, the purpose of this study is to explore the relationships among smoking resistance self-efficacy, trait coping style and nicotine dependence.

## METHODS

This cross-sectional study is from the baseline survey data of National Key R&D Program of China (2017YFC1309400): ‘Study on the Optimization of Tobacco Dependence Management Model Based on Hospitals and Communities’. The survey subjects (n=692) were smokers who were willing to quit smoking in 19 communities in Beijing between 2018 and 2019. Inclusion criteria were based on: the World Health Organization’s definition of smoking of a current smoker (smoking >1 cigarette per day for 6 consecutive or cumulative months); being a current occasional smoker (smoking cigarettes >4 times per week, but <1 cigarette per day on average); being failed smoking quitters and meeting the smoking standards (have given up smoking but resumed smoking, or had not smoked for <2 years at the time of the survey); being an ex-smoker, smoking every day for at least 6 months, but no longer a smoker at the time of the survey^18^. All participants performed a CO test to verify whether they smoked or not. After the CO test, smokers whose carbon monoxide score was greater than 0 were included in this study. Participants who had quit smoking or scored 0 on the carbon monoxide test were excluded. Subjects were screened and questionnaires were reviewed according to the inclusion exclusion criteria, and 568 valid questionnaires were retained. This project was reviewed and approved by the Ethics Committee of Capital Medical University (Batch number: Z2019SY007).

### Research tools

The questionnaire used in this study includes four parts: sociodemographic questionnaire, Fagerström test of nicotine dependence (FTND), smoking abstinence self-efficacy (SASE), and trait coping style questionnaire (TCSQ).

#### Sociodemographic questionnaire

Sociodemographic characteristics were obtained including age, gender, marital status, education level, average monthly income level, household registration nature, work status, and other information.

#### Fagerström test of nicotine dependence (FTND)

The scale consists of 6 items, each item is scored on a scale of 0–3, representing a scale from least to most dependent. The total score of 6 items was used to judge nicotine dependence, which was divided into 3 levels: mild 0–3; moderate 4–6; and severe >6. Cronbach’s coefficient of the Chinese version of the nicotine dependence scale was 0.658^19^, indicating that its internal consistency reliability was fair.

#### Smoking abstinence self-efficacy (SASE)

A hierarchical model that integrates the previously competing models provided the best fit to the data and served to explain a large body of previous findings. The model includes three first order constructs (Positive/Social; Negative/Affective; and Habit/Addictive)^9^. The model was used to measure the sense of self-efficacy in refusing to smoke in different smoking triggering situations, i.e. whether the individual can be convinced that he or she is in control of not smoking in the smoking triggering situation. The scale included three situational dimensions and consisted of nine items to measure the smoking abstinence self-efficacy of the study subjects. The scale is based on a 5-point Likert scale: 1=Extremely want to smoke, 2=Very want to smoke, 3=Some want to smoke, 4=Not much want to smoke, and 5=Do not want to smoke at all. The higher the score, the higher the level of smoking abstinence self-efficacy. The Cronbach’s coefficient of the scale is 0.884, indicating a good internal consistency reliability^9,20^. The scale has three dimensions: positive/social context dimension; negative/emotion context dimension, and habit/addiction context dimension.

#### Trait coping style questionnaire (TCSQ)

Coping is an important mediator of the psychological stress process and is related to both the nature of the stress event and the stress outcome. For example, from the perspective of the subject of coping activities, coping involves individual psychological activities (e.g. re-evaluation), behavioral operations (e.g. avoidance) and somatic changes (e.g. relaxation); from the relationship between coping activities and the stress process, coping involves various aspects of stress, including life events (e.g. confrontation, avoidance, problem solving), cognitive evaluation (e.g. self-blame, fantasy, downplaying), social support (e.g. asking for help, confiding, isolation) and psychosomatic responses (e.g. relaxation, smoking, alcohol, taking medication); from the point of view of the direction of coping activities, there are problem-specific coping and emotional coping; and so on^21^.

The trait coping style questionnaire collects, to the extent possible, the content of a variety of relatively stable coping behaviors or cognitive activities obtained in the psychopathological etiology survey, based on the content of psychological defence mechanisms, and uses them as a basis for entries: by repeated pretesting, screening those entries that are related to stress response effector variables such as SCL-90 and finally by factor screening to determine positive and negative coping categories. The responses formed in this way have the following characteristics: 1) there is a certain consistency across scenarios; 2) they are related to certain personality traits; 3) they are related to mental and physical health; and 4) as a result of the first three, the content of each entry is focused on emotional responses. In summary, the response items on this questionnaire reflect the trait attributes that individuals have and the health-related coping styles, and are therefore called trait coping questionnaires^21^.

This scale contains 20 items in total, and adopts the Likert 1–5 point scoring method, including negative coping (NC) and positive coping (PC). Cronbach’s coefficients of the whole scale and the two subscales were 0.90, 0.89 and 0.78, respectively, indicating good internal consistency. This scale measures individual trait attributes, which are related to health-related coping styles^21^.

### Survey methods and quality control

In order to prevent the respondents from giving wrong cognition and feedback to the questions they were asked during the investigation, all on-site investigators were given collective training. At the same time, the quality review and double-record were carried out after the completion of the investigation, to ensure the authenticity of the investigation information.

### Statistical methods

Epidata was used to establish and manage the database, and the collected data were imported into SPSS22.0 (IBM SPSS Statistics) for analysis. Nicotine dependence scores and psychological trait scores were examined under different demographic information using Analysis of Variance. Spearman Correlation Analysis was used to examine the correlation between nicotine dependence and factors. Validation was tested using the Process macro program written by Hayes (Process is a plug-in in the SPSS program, the plug-in can be used to test for mediating effects); the Boostrap algorithm was used for path analysis among nicotine dependence, smoking rejection self-efficacy and coping style; and the Bootstrap method was used for mediating effect test. A value of p<0.05 was considered statistically significant. Mediation is an important methodological concept in social science research. If the independent variable X has a certain influence on the dependent variable Y through a certain variable M, then M is called the intermediate variable of X and Y. The purpose of mediating effect analysis is to determine whether the relationship between the independent variable X and the dependent variable Y is partly or entirely attributable to the mediating variable M^22^.

## RESULTS

The majority of respondents in this survey were male (90.0%), the majority (63.6%) were middle-aged and older than 50 years old, marital status was dominated by married status (86.3%), college or Bachelor’s degree (39.9%) and senior high school/technical secondary school/technical school (28.7%) were the majority in education level, the type of work is consistent with age characteristics with the majority retirees (41.9%), the average monthly income in RMB (1000 Chinese Renminbi about 150 US$) was mainly in the ranges 2001–4000 RMB (27.8%) and 4001–6000 RMB (27.8%), and most were urban registered permanent residents (84.7%).

### SASE of the study subjects

There was a significant difference in the habit/addiction dimension in the SASE between different genders (p=0.029), with men (2.50±0.89) scoring significantly lower than women (2.78±1.23) on this dimension. There were significant differences in the negative/emotional situation dimensions in the SASE among different age groups (p<0.001), the smoking abstinence self-efficacy score of people aged ≥50 years (2.39±1.05) in the negative/emotional situation was significantly higher than that of people aged 30–50 years (p=0.002, p=0.001). There was a significant difference in the SASE in negative/emotional situations among respondents with different educational levels (p=0.007), the score of respondents with a junior high school education (2.50±1.08) was significantly higher than that of those with a junior college education (2.14±0.84; p=0.014) or a postgraduate education (1.89±0.75;p=0.018). Smokers with different job types had significantly different smoking abstinence self-efficacy in the negative/emotional context dimension (p<0.001), the smoking abstinence self-efficacy of retired employees (2.46±1.06) was significantly higher than that of commercial and service employees (1.95±0.84; p<0.001) and professional technicians (1.94±0.78; p=0.001) in the dimension of negative/emotional situations.

Smokers with different average monthly income had significant differences in SASE in the dimension of negative/emotional situations (p=0.021); smokers with an average monthly income of 8001–10000 RMB and >10000 RMB had significantly lower smoking abstinence self-efficacy (Table 1).

**Table 1:** Comparison of the score of SASE differences among participants in 19 communities in Beijing in 2019 with different sociodemographic characteristics (N=568)

*Characteristics*	*Categories*	*Number*	*Total score of smoking abstinence self-efficacy Mean±SD*	*p*	*Habit/addiction scenario dimension Mean±SD*	*p*	*Negative/emotional situation dimension Mean±SD*	*p*
**Gender**	Male	511	2.42±0.71	0.215	2.50±0.89	0.029	2.27±0.98	0.779
	Female	57	2.55±0.98		2.78±1.23		2.23±1.15	
**Age** (years)	<30	30	2.44±0.68	0.067	2.80±0.96	0.0612	1.88±0.70	<0.001
	30–50	177	2.33±0.65		2.46±0.84		2.08±0.87	
	>50	361	2.49±0.78		2.53±0.96		2.39±1.05	
**Level of education**	Primary school and below	40	2.29±0.74	0.082	2.28±0.81	0.417	2.29±0.10	0.007
	Junior high school	120	2.58±0.77		2.61±0.90		2.50±1.08	
	High school or other^a^	163	2.44±0.83		2.54±1.00		2.32±1.11	
	College or Bachelor’s	223	2.40±0.66		2.52±0.92		2.14±0.84	
	Postgraduate or above	22	2.26±0.50		2.42±0.79		1.89±0.75	
**Type of work**	Production, operation and service personnel	63	2.43±0.77	0.006	2.60±0.96	0.229	2.29±0.97	<0.001
	Business and service personnel	77	2.23±0.63		2.30±0.86		1.95±0.84	
	Personnel of state institutions^c^	55	2.34±0.63		2.44±0.84		2.16±0.93	
	Professional technician	57	2.25±0.61		2.47±0.86		1.94±0.78	
	Other workers^b^	78	2.52±0.72		2.56±0.81		2.32±1.01	
	Retired person	238	2.54±0.80		2.59±1.01		2.46±1.06	
**Average monthly income** (RMB)	<2000	48	2.44±0.87	0.189	2.44±0.97	0.543	2.48±1.17	0.021
	2001–4000	158	2.49±0.76		2.57±0.87		2.37±1.05	
	4001–6000	158	2.48±0.76		2.58±0.99		2.33±0.99	
	6001–8000	91	2.47±0.70		2.54±0.92		2.19±0.96	
	8001–10000	55	2.24±0.72		2.32±0.93		1.98±0.87	
	>10000	58	2.30±0.56		2.51±0.89		2.04±0.73	

a Others including technical secondary school and technical school.

b Including soldiers, unemployed, students etc.

c Including state organs, party and mass organizations, enterprises and other institutions. SD: standard deviation. RMB: 1000 Chinese Renminbi about 150 US$.

### TCSQ of the study subjects

There was no statistically significant difference in negative coping styles among different demographic characteristics. Smokers of different ages had statistically significant differences in positive coping style scores (p<0.001); the positive coping style score of smokers aged >50 years (38.02±7.40) was significantly higher than that of smokers aged <30 years (32.97±6.05; p<0.001) or aged 30–50 years (35.04±7.49 ; p <0.001).

Smokers with different educational levels had significant differences in positive coping scores (p=0.002), with lower scores for positive coping styles among the respondents who had a college or Bachelor’s education and postgraduate education. Smokers with different job types showed significant differences in positive coping scores (p=0.008), and further showed that the positive copying style score of the retired personnel (38.26±7.63) was significantly higher than other occupational groups (Table 2).

**Table 2:** Results of 2019 Beijing 19 community participants’ positive coping styles score comparison with different sociodemographic characteristics (N=568)

*Characteristics*	*Categories*	*Number*	*Positive coping style Mean±SD*	*F*	*p*
**Age** (years)	<30	30	32.97±6.05	14.10	<0.001
	30–50	177	35.04±7.49		
	>50	361	38.02±7.40		
**Level of education**	Primary school and below	40	36.68±6.31	4.41	0.002
	Junior high school	120	38.63±7.58		
	High school or other^a^	163	37.42±7.93		
	College or Bachelor’s	223	35.81±7.08		
	Postgraduate or above	22	33.14±8.39		
**Type of work**	Production, operation and service personnel	63	36.16±7.92	3.18	0.008
	Business and service personnel	77	35.38±7.58		
	Personnel of state institutions^c^	55	36.04±7.61		
	Professional technician	57	36.07±6.05		
	Other workers^b^	78	35.51±7.26		
	Retired person	238	38.26±7.63		

a Others including technical secondary school and technical school.

b Including soldiers, unemployed, students etc.

c Including state organs, party and mass organizations, enterprises and other institutions. SD: standard deviation.

### FTND of the subjects

There were significant differences in FTND among smokers with different educational levels (p=0.031), the score of the subjects with a primary school education (5.13±2.36) was significantly higher than that of those with a high school education/technical secondary school education (4.19±2.55; p=0.033) and those with a college education (4.04±2.40; p=0.011). The scores of people with a junior high school education (4.73±2.60) were significantly higher than those with a junior college education or a Bachelor’s degree (4.04±2.40; p=0.015). But there was no significant difference in FTND among other demographic characteristics.

### Nicotine dependence, smoking abstinence self-efficacy, and trait coping style

The results in Table 3 suggest that there was a significant negative relationship between FTND score and SASE total score (r=-0.494; p<0.05), positive/social dimension (r=-0.346; p<0.05), negative/emotional dimension (r=-0.555; p<0.05) and habit/addiction dimension (r=-0.555; p<0.05) of SASE. The negative coping style score was significantly positively correlated with the FTND score (r=0.208; p<0.05) and the negative coping style were significantly negatively correlated with the SASE total score, negative/emotional scenario dimension and habit/addiction dimension.

**Table 3:** Correlation matrix of smoking abstinence self-efficacy, coping style and nicotine dependence

	*1*	*2*	*3*	*4*	*5*	*6*	*7*
**1 Nicotine dependence score**	1	-	-	-	-	-	-
**2 Total score of smoke abstinence self-efficacy**	-0.494**	1	-	-	-	-	-
**3 Positive/social context dimension**	-0.346**	0.791**	1	-	-	-	-
**4 Negative/emotional situation dimension**	-0.299**	0.811**	0.449**	1	-	-	-
**5 Habit/addiction scenario dimension**	-0.555**	0.824**	0.525**	0.473**	1	-	-
**6 Negative coping style**	0.208**	-0.171**	-0.030	-0.226**	-0.141**	1	-
**7 Positive coping style**	-0.066	0.050	0.007	0.117*	-0.011	-0.109*	1

* p<0.05.

** p<0.01.

Since there was no significant correlation between positive coping style and FTND and SASE scale, and no significant correlation between demographic variables and FTND, positive coping style and demographic information variables were not considered in the verification of the intermediary analysis. FTND, total SASE, negative/emotional situational score, habit/addiction situational score, and negative coping style found in the correlation analysis were incorporated into the pathway.

The results in Table 4 show that there was a significant positive correlation between negative coping style and FTND (β=0.13; p=0.0003), and a significant negative correlation between SASE in the habit/addiction situation and FTND (β=-0.53; p<0.001), However, SASE in the dimension of negative/emotional situation had no significant score on nicotine dependence, so in the path of SASE and nicotine dependence in the dimension of negative coping style, the negative/emotional situation was excluded from the mediation analysis.

**Table 4:** Regression analysis of variables in the model

*The regression equation*		*Global fit index*		*Significance of regression coefficient*	
*The results of variable*	*Predictor variable*	*R^2^*	*F*	*β*	*t*
Negative/situational dimension smoking abstinence self-efficacy	Negative coping style	0.05	30.37***	-0.23	-5.51***
Habit/addiction dimension smoking abstinence self-efficacy	Negative coping style	0.02	11.50***	-0.14	-3.39***
Nicotine dependence score	Negative/situational dimension smoking abstinence self-efficacy			-0.02	0.50
	Habit/addiction dimension smoking abstinence self-efficacy	0.57	90.59***	-0.53	-13.41***
	Negative coping style			0.13	3.63***

All variables in the model are put into the regression equation after standardized treatment.

* p<0.05.

** p<0.01.

*** p<0.001.

**Figure 2:**
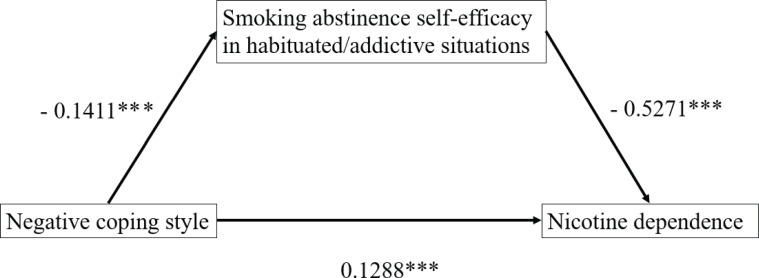
Mediating effect pathway between negative coping style and nicotine dependence of smoking abstinence self-efficacy in habit/addiction situation

The analysis of the mediating effect of smoking abstinence self-efficacy in the habit/addiction situation between negative coping style and nicotine dependence shows that the indirect effect of smoking abstinence in the habit/addiction situation self-efficacy in the influence of negative coping style and nicotine dependence was 0.08, and the 95% CI of Bootstrap does not include 0, which indicates that smoking abstinence self-efficacy in habituated/addictive situations has a significant mediating effect between negative coping style and nicotine dependence (Table 5 and Figure 2).

**Table 5:** Mediating effect analysis of smoking abstinence self-efficacy between negative coping style and nicotine dependence in habit/addiction situation of smokers in 19 communities in Beijing in 2019

*Effect*	*Value of effect*	*Boot standard error*	*Boot CI lower limit*	*Boot CI upper limit*	*Relative mediation effect*
**Total**	0.21	0.04	0.12	0.29	-
**Direct**	0.13	0.04	0.06	0.20	62.01 %
**Indirect**	0.08	0.03	0.03	0.13	37.99 %

The standard error of Boot, the standard error of Boot CI lower limit and the standard error of Boot CI upper limit of indirect effect estimated by the percentile Bootstrap method of deviation correction, and the lower limit and upper limit of 95% confidence interval (CI), respectively.

The results showed that individuals who used more passive coping styles had higher scores for nicotine dependence (β=0.13; p<0.001). That is, in the face of stress and stress situations, individuals who choose negative coping styles, such as escape and unhealthy lifestyle, will produce more smoking behaviors, thus increasing their dependence on nicotine. At the same time, smoking abstinence self-efficacy in the face of habit-addiction situations mediated indirectly the effect of negative coping styles on nicotine dependence, that is, more negative coping styles will lead to quitters who do not think they can resist the temptation to smoke when faced with some smoking habit situations, such as getting up in the morning and after dinner. In other words, it means a decrease in self-efficacy (β=-0.14; p<0.001); a decrease in smoking abstinence self-efficacy leads to an increase in nicotine dependence (β=-0.53; p<0.001). Further information is also given in the Supplementary file.

## DISCUSSION

Research on methods and approaches to smoking cessation has developed in China as awareness of the hazards of smoking and the dangers of secondhand and thirdhand smoke have spread, which shows that the Chinese Government has realized the importance of smoking cessation^23^, especially with the development of information technology^24^, and more and more smokers who want to quit are trying electronic information system interventions for smoking cessation methods and approaches^25^. However, we still find that most of the concerns about the impact factors of smoking cessation focus on the perception of smoking harm and smoking cessation drugs usage instead of psychological intervention. As we know, tobacco dependence, alcohol dependence and drug dependence have common characteristics, and physical changes are accompanied by psychological characteristics. Therefore, attention to the psychological characteristics of ordinary smokers is an important part of improving smoking cessation intervention in the future. Our study explored the characteristics and relationships among smoking abstinence self-efficacy, trait coping style and nicotine dependence of smokers who want to quit smoking, and provided a theoretical basis for the intervention of quitters.

According to the total score of smoking abstinence self-efficacy, retired people have the highest smoking abstinence self-efficacy, significantly higher than the commercial, service personnel and professional technicians. On the one hand, most retired people are older, and the older they are, the easier they find it to quit smoking, which is consistent with previous studies^26,27^. On the other hand, commercial and service personnel have a high degree of freedom in their work, and most of them work in the context of human interaction. Under the influence of tobacco and alcohol culture, they often gather with people to smoke because of the nature of their work^28^. The willingness of professional and technical personnel to quit smoking is generally low, which is consistent with previous studies^29^. Most of their work is repetitive and requires a high level of concentration, which stimulates nicotine craving. From the smoking abstinence self-efficacy in habit/addiction dimension, the score of men is significantly lower than that of women, which is consistent with the feature that men smoke much more than women, so the degree of addiction is more serious. Analyzing the characteristics of people who were willing to quit smoking on the negative/emotional scale, it was found that young and middle-aged people had lower scores; relevant studies have shown that emotional experiences and social situations can lead to smoking behaviors^30^; young and middle-aged people are more likely to face the pressure of work and life, so smoking abstinence self-efficacy is lower in this dimension. Those with a graduate degree or above, a Bachelor’s degree or an associate’s degree scored significantly lower than those with a junior high school degree in this dimension; the people with higher education are engaged in more complex mental work, the competition in the workplace and the continuous innovation of new technologies and methods make those with higher education to face greater work pressure and competitive pressure. At the same time, social need is also an important factor influencing smoking behavior^31^. The work status was consistent with the total score of smoking abstinence self-efficacy. The monthly income level is consistent with the relevant characteristics of education level.

Young and middle-aged people who want to quit smoking have lower scores for positive coping styles and therefore are more likely to use smoking as a way of dealing with stressful events; the reason for this phenomenon can be attributed to the fact that older people have more experience and better emotional control ability and are more likely to choose the coping style of problem solving; young people, on the other hand, are more impulsive and therefore more likely to smoke during stressful events. People with college, undergraduate and graduate degrees who want to quit smoking have lower scores for positive coping strategies. However, highly educated people are aware of the dangers of smoking; they are more likely to choose smoking in the face of job competition pressure, with higher the risk of addiction^31^. The positive response score of retired personnel is significantly higher than that of production operators, commercial personnel, service personnel, personnel of state organs, party and mass organizations, enterprises and institutions, professional and technical personnel, and other labor personnel. On the one hand, age influences coping style, on the other, the nature of work determines exposure to tobacco products, and these factors determine if people are more likely to use smoking as a way to cope with stress after becoming addicted.

The results showed that individuals who used more negative coping styles were more likely to engage in smoking behavior and so were more nicotine dependent; the use of negative coping style will reduce the self-efficacy of refusing smoking under the influence of addiction, thus leading to the generation of smoking behavior. This can be explained by learned helplessness. Learned helplessness refers to a hopeless and negative psychological state of reality acquired through repeated failure and frustration^32^, as opposed to a belief in self-efficacy, therefore, higher self-efficacy, that is, higher confidence and effort level that can help individuals face stressful events^33^. So, when an individual has a belief in the addictive situation that he or she can stop smoking, it counteracts some of the negative coping style’s effect on nicotine dependence.

### Strengths and limitations

In this study, two psychological traits were selected to influence smoking cessation behavior, smoking abstinence self-efficacy and trait coping, and used to analyze the groups of people to focus on. In combining the two traits with the degree of nicotine dependence to explore the relationship between the three, the mediating role of self-efficacy in smoking rejection was validated, and the relationship between the two psychotropic effects and nicotine dependence was characterized more clearly.

There are still some limitations in this study. First, the sample size of this study was only 19 communities in Beijing, which failed to include smokers from other areas and did not explore geographical characteristics. Second, the study is based on a cross-sectional study, which cannot completely confirm the dynamic relationship between the psychological traits of smokers and their nicotine dependence. Therefore, prospective cohort studies are needed to verify the results.

## CONCLUSIONS

This study found that self-efficacy and coping style of the groups with the intention to quit smoking was influenced by age, educational background and occupational type; self-efficacy played an indirect mediating role between the negative coping style and nicotine dependence, individuals who used more negative coping styles were more likely to engage in smoking and, therefore, were more nicotine dependent. When providing smoking cessation intervention and counselling to smokers who have the intention to quit smoking, it is necessary to reduce the use of negative coping strategies and improve the self-efficacy of smoking abstinence in the face of addiction. First, there is a need to cultivate the healthy behavior habits of quitters, and to teach them to use alternatives and behaviors in the face of addiction situations such as smoking cravings. Second, there is a need to train quitters to use correct coping methods in the face of pressure, stress events and other adverse circumstances, to reduce the occurrence of smoking-based escape methods. Finally, more attention should be focused on the intervention of young and middle-aged people and the people who work in commercial services and professional technology to quit smoking.
