# Supplementary material for: Relationships among smoking abstinence self-efficacy, trait coping style and nicotine dependence of smokers in Beijing
Source: Tob Induc Dis. 2020 Sep 1;18:72. doi: 10.18332/tid/125401 (PMC7485440; doi:10.18332/tid/125401)
Supplement: Supplementary file 1 [file TID-18-72-S1.pdf]

Table 1 Dimensions of Smoking Abstinence Self-Efficacy Scale

Dimension	Items and contents
Positive Smoking Abstinence Self-Efficacy,SASEP	○,1 At parties with friends; ○,2 Tea, rest, chat;○,3 Spouses, or good friends are smokers when with them;
Negative Smoking Abstinence Self-Efficacy,SASEN	○,1 When feeling more anxious and depressed;○,2 When very angry;○,3 When things don't go your way or you fail;
Habitual Smoking Abstinence Self-Efficacy,SASEH	○,1 After getting up in the morning;○,2 When you feel you need refresh yourself;○,3 When you think you haven't smoked in a while

Table 2 Demographic Information of Respondents (n=568)

Demographic information	The grouping	Number of people	The percentage （%）
Gender	Male	511	90.0
	Female	57	10.0
Age	Under the age of 30	30	5.3
	Age of 30-50	177	31.2
	Age 50 and above	361	63.6
Marital status	Unmarried	39	6.9
	Married	490	86.3
	Divorced	21	3.7
	Others (separation, widowhood, etc.)	18	3.2
Level of education	Primary school and below	40	7.0
	Junior high school	120	21.1
	High school/technical secondary school/technical school	163	28.7
	College or bachelor	223	39.3
	Postgraduate or above	22	3.9
Type of work	Production, operation and service personnel	63	11.1
	Business and service personnel	77	13.6
	Personnel of state organs, party and mass organizations, enterprises and institutions	55	9.7
	Professional technician	57	10.0
	Other workers (soldiers, unemployed, students, etc.)	78	13.7
	Retired person	238	41.9

Average monthly income	2000 yuan or less	48	8.5
	2001-4000 yuan	158	27.8
	4001-6000 yuan	158	27.8
	6001-8000 yuan	91	16.0
	8001-10000 yuan	55	9.7
	10,000 yuan and above	58	10.2
Account type	Urban registered permanent residence	481	84.7
	Agricultural registered permanent residence	87	15.3
Total		568	100

Table 3 Comparison of Nicotine Dependence Scores Among Different Demographic Information (n=568)

Demographic indicator	Group	Nicotine dependence score	*F*	*p*
Level of education	Primary school and below	5.13±2.355	2.686	0.031
	Junior high school	4.73±2.602		
	High school/technical secondary school/technical school	4.19±2.545		
	College or bachelor	4.04±2.402		
	Postgraduate or above	4.32±2.124		

*p<0.05 was statistically significant.

© 2020 Ma H. et al.
